# Impact of the COVID-19 Pandemic on the Perceived Quality of Palliative Care in Nursing Homes

**DOI:** 10.3390/jcm11195906

**Published:** 2022-10-06

**Authors:** Emilio Mota-Romero, Concepcion Petra Campos-Calderon, Daniel Puente-Fernandez, Cesar Hueso-Montoro, Ana A. Esteban-Burgos, Rafael Montoya-Juarez

**Affiliations:** 1 Dr. Salvador Caballero García Primary Care Centre, Andalusian Health Service, Government of Andalusia, 18012 Granada, Spain; 2 Doctoral Programme in Clinical Medicine and Public Health, University of Granada, 18071 Granada, Spain; 3 Department of Nursing, University of Granada, 18071 Granada, Spain; 4 Instituto de Investigación Biosanitaria, 18012 Granada, Spain; 5 Community Mental Health Unit, Andalusian Health Service, Government of Andalusia, 18800 Baza, Spain; 6 Department of Nursing, University of Jaen, 23071 Jaen, Spain; 7 Instituto Mente, Cerebro y Comportamiento (CIMCYC), University of Granada, 18016 Granada, Spain

**Keywords:** palliative care, palliative medicine, nursing homes, COVID-19, SARSCOV2, primary care

## Abstract

The Nursing Homes End-of-life Programme (NUHELP) was developed in 2017 and is based on quality standards of palliative care, but it was not implemented due to the outbreak of the COVID-19 pandemic. Objectives: To describe perceptions among staff at nursing homes and primary health care (PHC) centres regarding the relevance, feasibility, and degree of achievement of quality standards for palliative care in nursing homes and to determine the differences in these perceptions before and after the pandemic. Methodology: Cross-sectional descriptive study. Professionals at eight nursing homes and related PHC centres who participated in NUHELP development assessed 42 palliative care standards at two time points (2018 and 2022). The Mann–Whitney U test was applied to analyse differences in the scores between these two times and between perceptions at nursing homes and at PHC centres. Results: The study population consisted of 58 professionals in 2018 and 50 in 2022. The standard regarding communication with persons affected by the death of a family member was considered less relevant (*p* = 0.05), and that concerning the culturally sensitive and dignified treatment of the body was less fully achieved (*p* = 0.03) in 2022 than in 2018. Social support (*p* = 0.04), sharing information among the care team (*p* = 0.04), patient participation (*p* = 0.04) and information about the treatment provided (*p* = 0.03) were all more poorly achieved in 2022 than in 2018. The perceptions of nursing home and PHC workers differed in several respects. Conclusions: Professional intercommunication and social support should be reinforced, and residents should be more actively involved in decision-making.

## 1. Introduction

The global population is ageing. According to the World Health Organization, by 2050, there will be twice as many people aged over 65 as in 2019 (rising from 0.7 to 1.5 billion) [1]. By 2068, people aged over 65 years will constitute 29.4% of the Spanish population, compared to the 19.1% recorded in 2018 [2]. Spain will have the fourth highest ratio of dependency and of older people in the European Union [3].

In the future, there will be a greater need for caregivers for older people with chronic diseases. The ability of families to care for the elderly is conditioned by the transformation of family life, decreasing fertility and rising migration to urban areas, with the consequent geographical dispersion, among other factors [4].

In response to the foreseeable shortfall of informal caregivers, more older people will live in institutions. As an indication of this trend, the number of persons residing in nursing homes rose by 4.56% between 2015 and 2020 [5].

For many older people, nursing homes are their last place of life, and deaths in these institutions are expected to double by 2040 [6]. This outlook highlights the importance of the palliative care provided in nursing homes, where there is a high prevalence of chronic diseases [7]. An estimated 60–70% of chronic patients with palliative needs are residents in nursing homes [8].

Recent studies in Spain and the EU indicate that many residents of nursing homes receive unnecessary interventions at the end of life, such as cardiopulmonary resuscitation, mechanical ventilation, surgery, dialysis, blood transfusions, chemotherapy or radiotherapy [9,10,11,12,13]. The provision of high levels of medication is also common in the last weeks of life, and not all are related to symptom control [11,13].

Various institutions have emphasised the need for nursing home personnel to receive comprehensive training in end-of-life care [14,15] via programmes such as the Gold Standards Framework [16] or Namaste Care [17], which is specifically designed for nursing home residents with dementia.

In Spain, a team of researchers from Granada and Jaén Universities developed the Nursing Home End of Life Programme (NUHELP) in 2017 to improve end-of-life care in nursing homes [18].

This programme is based on 42 standards for end-of-life care in adult palliative care units, published by the British National Institute for Health and Care Excellence [19] and the New Health Foundation [20].

These standards were evaluated in terms of their relevance, feasibility and degree of implementation at participating nursing homes and the health centres with which they were associated [18].

The COVID-19 pandemic provoked a radical change in the care provided by nursing homes, which were forced to adapt their internal operations in order to protect residents and staff from contagion [21]. Among the new measures adopted, family visits were restricted, access was controlled (with obligatory temperature testing and entry/exit registration), strict hand hygiene was required, the respiratory symptoms of staff and residents were assessed, and government-mandated protocols in response to positive contacts were applied [22].

Despite these measures, the mortality in nursing homes remains unacceptably high. In Spain, there have been 30,240 deaths from the SARS-CoV-2 virus in nursing homes since the start of the pandemic [23]. This mortality has been aggravated by demographic and healthcare-related factors (such as community life and the lack of personal protective equipment), regulatory and administrative factors regarding employment practices, and by factors related to comorbidity [24,25].

In view of these circumstances, the following question arises: do the staff at the nursing homes and primary health care (PHC) centres participating in the NUHELP programme attach the same value today as in 2018 to the quality standards then proposed for palliative care, or have their perceptions changed following the experience of the pandemic?

Among the barriers to the provision of end-of-life care in nursing homes, responsibilities often remain unidentified, and coordination among the professionals involved is inadequate or non-existent [26]. Good communication is of vital importance, enabling mutual support between nursing home staff and those working in PHC [27]. Indeed, one of the strengths of the NUHELP programme is that it was designed jointly by nursing home and PHC personnel. However, there may be differences between these professionals in their perceptions of the quality of palliative care.

The aims of this study are, on the one hand, to describe the relevance, feasibility and degree of achievement perceived by the nursing home and PHC staff of quality standards for palliative care in nursing homes before and after the COVID-19 pandemic and, on the other, to detect and identify any differences among the perceptions of those working in these two contexts.

## 2. Materials and Methods

### 2.1. Design

This cross-sectional descriptive study was carried out at two time points (2018 and 2022). It cannot be considered a prospective study since the participants varied between the two periods.

In the following sections, the participants and the data collection procedure are described for each time point considered. The instruments used and the data analysis procedures were identical in each case.

### 2.2. Sample

The study was conducted among personnel at the eight nursing homes and PHC centres which collaborated in the development of the NUHELP programme. These institutions were chosen according to three criteria:

- That the staff at the nursing homes and PHC centres involved were willing to participate in the development and subsequent implementation of the programme.
- That the nursing homes had contracted with the local social services for the provision of assistance to the elderly.
- That the nursing homes catered for at least 60 residents (this stipulation arose from the fact that centres of this size are required to have nursing staff on call 24 h a day and an in-house doctor).

In 2018, an intentional sampling was carried out, recruiting 58 healthcare professionals (in the fields of nursing, medicine, social work and psychology) from nursing homes and PHC centres (medicine and nursing) associated with the NUHELP programme [18].

For the 2022 study, in addition to the aforementioned criteria, an additional requirement was included that the participants should have worked in these centres during the first waves of the COVID-19 pandemic (March–November 2020).

### 2.3. Data Collection Procedure

In 2018, members of the NUHELP programme visited the participating centres to inform them of the Project’s aims and to request the participation of their staff. Once consent had been obtained, the project researchers contacted potential staff members by email, inviting them to return an online form. This data collection process took place from January to March 2018.

In 2022, these same staff members were again contacted and asked to complete the same form again. In addition, the contact persons at each centre were asked to forward the form to other personnel who met the inclusion criteria and might be interested in taking part. This data collection process was carried out from January to March 2022.

### 2.4. Tools 

The questionnaire developed ad hoc for this study addressed the following sociodemographic and clinical variables: age, sex, professional category, employment pattern (full-time/part-time) and setting (nursing home/PHC centre).

The questionnaire also included the 42 quality standards incorporated into the NUHELP programme, ordered by contextual relevance and detailed in Annex I.

Each participant was asked to rate the standards according to the following criteria and scored on a Likert scale ranging from 1 (totally disagree) to 5 (totally agree).

- Appropriateness of the care provided in the nursing home in question.
- Feasibility of its implementation of palliative care.
- Level of attainment, i.e., the extent to which the recommended palliative care was provided.

The third criterion was only evaluated for the nursing homes since it was assumed that PHC personnel could not be expected to accurately determine this question for their place of work.

### 2.5. Statistical Analysis

The continuous study variables were analysed using descriptive measures of central position and dispersion (mean and standard deviation) and the discrete variables by frequencies. The application of the Kolmogorov–Smirnov test showed that the responses to the different standards did not follow a normal distribution, and so the inferential analysis was performed by non-parametric tests.

Two-tailed Mann–Whitney U and chi-square tests were performed to determine whether there were significant differences between the samples for the variables collected regarding both the time points considered (2018 and 2022) and the two groups of personnel (at nursing homes and PHC centres). 

### 2.6. Ethical Considerations

The study was approved by the Research Ethics Committee (0706-N-17). Information about the project was included in the online questionnaire. All of the participants provided prior informed consent and their contact information. At all times these data were separated from the questionnaire answers in order to protect the participants’ anonymity, in accordance with Organic Law 3/2018, of 5 December, on Personal Data Protection and Safeguards for Digital Rights [28]. 

## 3. Results

### 3.1. Characteristics of the Sample

In total, 108 healthcare personnel participated in the study, 58 (53.7%) in 2018 and 50 (46.3%) in 2022. Only ten of those who took part in 2018 repeated their participation in 2022. Most of the 2018 participants who did not repeat in 2020 (*n* = 35) were due to changes of workplace, either due to the impossibility to contact them (*n* = 3) or refusal to participate again (*n* = 2). The mean age of the participants in 2018 was 41 years (*SD* = 10.7), while in 2022, it was 42 years (*SD* = 13.5). The total sample was composed of 84 women and 24 men. In 2018, these workers had overall professional experience and specific geriatric field experience of 10.9 years (*SD* = 8.4) and 11.2 years (*SD* = 7.7), respectively. In 2022, the corresponding figures were 11.3 years (*SD* = 7.8) and 14.2 years (*SD* = 9.78). The male:female ratio, the professional categories involved, the employment pattern and work settings were all similar at both time points. Table 1 describes the demographic data of the sample in greater detail.

### 3.2. Differences in Palliative Care Standards between 2018 and 2022 According to Relevance, Feasibility and Level of Attainment

With respect to the perceived relevance of palliative care standards, the participants awarded this criterion a score greater than four points (out of five) in both 2018 and 2022. The standard that received the highest score in 2018 was No. 40, “Communication with people greatly affected by the death of a family member is carried out in a sensitive manner” (4.71/0.59). In 2022, the highest score corresponded to No. 38, “The body of a person who has died is cared for in a culturally sensitive and dignified manner” (4.52/0.89). The lowest score in 2018 was for No. 32, “There is an education programme for patients and families who wish to use it, which facilitates decision-making throughout the course of the disease.” (3.97/1.15), while in 2022, it was for No. 14, “People approaching the end of life have their psychological needs safely, effectively, and appropriately met at any time of day or night, including access to medicines and equipment.” (3.94/1.24). Significant differences between the two time points were only found for No. 40, “Communication with people greatly affected by the death of a family member is carried out in a sensitive manner” (*p* = 0.05). In this case, the score in 2022 (4.44/0.81) was significantly lower than in 2018 (4.71/0.59). 

For the feasibility criterion, the highest-rated standard was No. 39, “Families and carers of people who have died receive timely verification and certification of the death.”, both in 2018 (4.64/0.61) and in 2022 (4.5/0.12). Those given the lowest scores were, in 2018, No. 32, “There is an education programme for patients and families who wish to use it, which facilitates decision-making throughout the course of the disease.” (3.24/1.23) and, in 2022, No. 2, “Generalist and specialist services providing care for people approaching the end of life and their families and carers have a multidisciplinary workforce sufficient in number and skill mix to provide high-quality care and support.” (3.30/1.16). 

Significant differences between the time points were only found for item No. 38, “The body of a person who has died is cared for in a culturally sensitive and dignified manner.” (*p* = 0.03), with significantly lower scores in 2022 (4.30/0.97) than in 2018 (4.62/0.77). 

With regard to the level of attainment, the highest rated standard in 2018 was No. 38, “The body of a person who has died is cared for in a culturally sensitive and dignified manner” (4.61/0.84), while in 2022, it was No. 39, “Families and carers of people who have died receive timely verification and certification of the death.” (4.24/1.08). The lowest rated standard was No. 32, “There is an education programme for patients and families who wish to use it, which facilitates decision-making throughout the course of the disease.”, both in 2018 (2.41/1.37) and in 2022 (2.60/1.34). 

For the level of attainment criterion, the scores for some standards were lower in 2022 than in 2018. This was the case for No. 15, “People approaching the end of life are offered social and practical support which is appropriate to their preferences and maximises independence and social participation for as long as possible.” (*p* = 0.045), No. 22, “Information about the patient is shared by all professionals involved in the care process.” (*p* = 0.044), No. 31, “The team enables the patient to be involved in decision-making throughout the course of the disease.” (*p* = 0.036) and No. 33, “The team provides information on the benefits and adverse effects of the treatments that may be provided to the patient.” (*p* = 0.026). Detailed results for these criteria are shown in Table 2.

### 3.3. Differences in Palliative Care Standards between Nursing Homes and Primary Health Care Professionals 

Some differences were observed in how health care professionals perceived the relevance and feasibility of the proposed standards according to their employment setting (nursing home or PHC centre) (Table 3).

Thus, for the relevance criterion, PHC professionals were awarded higher scores than their counterparts in nursing homes for the items that addressed the provision of a living will document for patients approaching the end of life (*p* = 0.045).

In relation to feasibility, nursing home staff were awarded higher scores than PHC personnel for four of the 42 standards, and notably so for No. 15, “People approaching the end of life are offered social and practical support which is appropriate to their preferences and maximises independence and social participation for as long as possible.” (*p* = 0.002) and No. 16, “People approaching the end of life are offered spiritual and religious support appropriate to their needs and preferences.” (*p* = 0.003).

These differences varied over time. In 2018, PHC professionals were awarded higher scores than nursing home staff regarding the existence of a living will document (*p* = 0.046) but lower ones regarding the presence of meeting rooms/offices/offices necessary for the performance of their care activity (*p* = 0.050). However, in 2022, there were no significant differences between the groups in the latter respect. 

In 2018, nursing home professionals were awarded higher feasibility scores than their PHC counterparts for many standards, especially No. 15, “People approaching the end of life are offered social and practical support which is appropriate to their preferences and maximises independence and social participation for as long as possible.” (*p* = 0.003), No. 16, “People approaching the end of life are offered spiritual and religious support appropriate to their needs and preferences.” (*p* = 0.003) and No. 17, “Families and carers of people approaching the end of life are offered comprehensive support in response to their changing needs and preferences.” (*p* = 0.002). However, these differences were not significant in 2022. On the other hand, differences were observed for standard No. 38, “The body of a person who has died is cared for in a culturally sensitive and dignified manner.”, which was considered more feasible by nursing home professionals than by those working in PHC centres (*p* = 0.027).

## 4. Discussion

The aim of this study is to consider how professionals working in nursing homes and associated primary health care centres perceived quality standards for palliative care, in terms of their relevance, feasibility and level of attainment, before and after the COVID-19 pandemic. In addition, we examine whether these two groups of professionals differ in their perceptions of the standards. 

Our results show that, before and after the outbreak of the COVID-19 pandemic, there were no major differences in how nursing home and PHC professionals perceived palliative care standards according to their relevance and feasibility. However, differences were observed in how they perceived the level of attainment of the standards. Moreover, there were significant differences between the nursing homes and PHC personnel in how they perceived the relevance and feasibility of the standards. 

Regarding the level of relevance assigned to each standard, it is noteworthy that No. 40, “People closely affected by a death are communicated with in a sensitive way.” obtained lower scores in 2022 than in 2018, although it was among the highest rated of all the standards, for both time points. 

The outbreak of the pandemic meant that the death of a family member had to be communicated remotely, rather than in person, a situation that was challenging for the professionals involved in different settings such as emergency services [29], nursing homes [30] and hospitals [31]. Adaptation to this new context was difficult for all concerned since facial expressions and non-verbal language became inaccessible [29]. In addition, the isolation of family members and/or severe restrictions on visits created a complex emotional situation that was very difficult to resolve telematically [30,31,32].

Regarding the feasibility criterion, in 2022, the professionals consulted considered it much less likely that the body of a person who had died would be cared for in a culturally sensitive and dignified manner. The protocols for handling the body of a person who has died from COVID-19 detail procedures for wrapping, transfer to the morgue, cold storage, autopsies, delivery and the necessary cleaning and environmental control, but other aspects of a psychological and culture are marginalised [33,34].

During the pandemic, images of bodies being stored in ice rinks or empty rooms [35,36] created in the public mind the view that persons who died from COVID-19 were being subjected to dehumanised treatment. This understanding would explain, at least in part, why health care professionals believe that respect for the body of the deceased is now more difficult to achieve than in 2018. 

The criterion ‘level of attainment’ generally received the lowest scores of the three, both in 2018 and 2022. This is consistent with previously available data on end-of-life care in nursing homes [21,37].

Our comparative analysis indicates that the mean values for all the standards were lower in 2022 than in 2018, which could reflect perceptions that the quality of care in nursing homes for persons approaching the end of life had deteriorated between the two time points. 

The decrease in social support in 2022 is particularly interesting because if anything has been affected by the pandemic, it is the social dimension of the end of life. Strang et al. [38] calculated that only 59% of those who died from COVID-19 were accompanied, either by professionals or family members. Despite the publication of regulations to prevent or limit the presence of relatives in nursing homes, the questionnaire responses indicated that these regulations were breached in order to facilitate the accompaniment of persons in their last days of life [39,40].

Additionally, observed in 2022 was a decrease in information sharing among professionals about their patients. This may have been because professionals felt confused by the constant changes in protocols on treatments, forms of transmission, adverse effects, etc. For example, in Spain alone, during the first wave of COVID-19 (12 March to 21 June 2020), 59 Official Bulletins related to COVID-19 were published [41]. 

During the first months of the pandemic, Miró et al. [42] conducted a survey of emergency departments and found that despite the protocols constantly being changed, more than 40% of emergency departments did not have a protocol in place for patients with severe COVID-19.

Other areas of attention that deteriorated during the pandemic were patient information services and decision-making. This outcome was closely related to the prevailing uncertainty regarding the evolution of the disease. A survey of the general population conducted by Köther et al. in 2020 [43] revealed a preference to participate in situations unrelated to COVID-19 (55.2%) than to take decisions related to the pandemic (39.9%). Furthermore, the absence of health care protocols on decision-making and the restrictions on visits by family members limited the scope for discussion of the patient’s wishes and of the risks and benefits of the guidelines for action and for reacting to new situations [44]. These factors, together with the above-described problem that difficult conversations had to be conducted telematically, represented major challenges to the decision-making process.

Our analysis revealed several differences between the perceptions of nursing homes and PHC professionals. Among the former, almost all of the standards are considered to be more feasible. This finding is in line with Guardia-Mancilla et al. [45], whose comparative study of perceptions of end-of-life care in nursing homes, hospitals and PHC centres concluded that nursing home staff were the most optimistic in this regard. Indeed, it is logical that those providing care in nursing homes should believe it easier to achieve recommended standards since they are more aware of their own resources. Another study conducted more than a decade ago [46], showed that, compared to nursing home personnel, external professionals working with hospice agencies considered many quality-of-life domains more difficult to address. This question should be investigated further, given the need to ensure fluid collaboration between nursing homes and PHC workers and thus provide adequate end-of-life care. The multiple experiences of collaboration between professionals in both fields during the pandemic have brought positions closer in this regard, enabling PHC personnel to learn more about the work carried out in nursing homes [40]. 

Although the sample is relatively small and heterogeneous, the professionals who participated in the study reflect the reality of nursing homes in Spain, with nurses being the largest group of health professionals working in these centres. Nursing home professionals tend to work as a team and share some of the same perspectives on patient care; therefore, we understand that their perceptions of the end of life might be similar. 

Furthermore, as reflected above, only 20% of the original sample repeated their participation in 2022, which made it impossible for us to conduct a prospective paired-sample analysis. On the other hand, this variation in the identity of respondents is understandable, as many nursing home workers have changed jobs recently, especially in 2020, in response to staffing problems in the public system.

Another limitation that needs to be considered is that this study is an exploratory study for which there is no specific measurement bibliography. Although the instrument used in this study has not been validated, the data collected can serve as a basis for future research, and it could be useful for stakeholders, administrators and health care professionals.

## 5. Conclusions

Our results suggest that there has been a certain deterioration in end-of-life care in nursing homes during the COVID-19 pandemic, especially in the level of attainment of quality standards for palliative care.

Regarding the relevance criterion, the results suggest that in 2022 less information was being made accessible in a sensitive way to family members after the death of residents than in the previous survey in 2018. 

As concerns feasibility, our results indicate that the professionals believed it less likely in 2022 that the body of a deceased person would be handled in a culturally sensitive and dignified way, compared to their views in 2018. 

Levels of attainment were believed to have fallen in all respects, particularly as concerns the sharing of information among professionals, the provision of social support for residents, the transmission of information to them and obtaining their active involvement.

Our results point to the urgent need to develop end-of-life intervention programmes in nursing homes. These programmes should pay special attention to communication between professionals, residents and with relatives, before and immediately after the death of the resident.

## Figures and Tables

**Table 1:** Characteristics of the Sample.

	2018 (*n* = 58)	2022 (*n* = 50)	*p*
Age (years):	41 (10.7)	42(13.5)	0.49 *
Professional experience (years):	10.9(8.4)	11.3(7.8)	0.45 *
Geriatric care experience (years):	11.2(7.7)	14.2(9.78)	0.15 *
Gender:			
Female	48 (82.8)	36 (72%)	0.25 **
Male	10 (17.2)	14 (28%)	
Qualification:			
Nurse	36 (62.1%)	37 (74%)	
Social worker	1 (1.7%)	2 (4%)	
Psychologist	9 (15.5%)	5 (10%)	
Physician	12 (20.7%)	4 (8%)	
Physiotherapist		2 (4%)	
Employment:			
Full time	48 (82.8%)	41 (82%)	0.918 **
Part time	10 (17.2%)	9 (18%)	
Work setting:			
Nursing home	37(63.8%)	21(42%)	0.33 **
Primary care	21(36.2%)	29(58%)	

* Mann–Whitney U test. ** Chi-Square.

**Table 2:** Differences in scores given by all participants between 2018 and 2022, according to relevance, feasibility and level of attainment.

			TOTAL				
			2018 *n* = 58 *		2022 *n* = 50 **		p ***
			M	SD	M	SD	
1	Health and social care workers have the knowledge, skills and attitudes necessary to be competent to provide high-quality care and support for people approaching the end of life and their families and carers.	R	4.43	0.84	4.14	0.93	0.077
		F	3.95	1.16	3.66	0.94	0.058
		A	3.80	1.09	3.36	1.01	0.118
2	Generalist and specialist services providing care for people approaching the end of life and their families and carers have a multidisciplinary workforce sufficient in number and skill mix to provide high-quality care and support.	R	4.38	0.91	4.00	1.18	0.096
		F	3.45	1.16	3.30	1.16	0.519
		A	3.50	1.09	2.96	1.16	0.147
3	The roles and competencies of all professionals on the palliative care team are clearly defined.	R	4.33	0.89	4.14	0.90	0.210
		F	3.83	1.19	3.78	1.07	0.615
		A	3.55	1.27	3.28	1.11	0.192
4	The interdisciplinary team coordinates and works together on case conferences for each of the cases treated by the team.	R	4.22	0.99	4.06	1.00	0.315
		F	3.50	1.25	3.66	1.22	0.432
		A	3.34	1.29	3.00	1.25	0.652
5	The criteria for referral to other professionals in the team/centre are clearly defined: criteria for care by the psychologist, criteria for care by the social worker, criteria for care by the counsellor or spiritual guide.	R	4.33	0.82	4.06	1.02	0.188
		F	3.83	1.11	3.54	1.16	0.208
		A	2.72	1.90	3.12	1.21	0.367
6	The clinical material and medication needed to carry out care work are available to staff.	R	4.29	0.99	4.36	0.94	0.780
		F	3.71	1.11	3.96	1.09	0.194
		A	3.61	1.02	3.70	1.15	0.409
7	The meeting rooms, offices, etc. necessary for carrying out care activities are available to staff.	R	4.47	0.78	4.32	0.91	0.456
		F	4.34	0.87	4.12	1.06	0.285
		A	4.20	0.95	3.82	1.12	0.113
8	People approaching the end of life are identified in a timely manner.	R	4.45	0.78	4.32	0.98	0.667
		F	4.14	0.85	3.90	1.11	0.399
		A	4.07	0.93	3.60	1.20	0.523
9	People approaching the end of life who may benefit from specialist palliative care are offered this care in a timely manner appropriate to their needs and preferences, at any time of day or night.	R	4.33	0.96	4.24	0.98	0.553
		F	3.74	1.04	3.56	1.07	0.387
		A	3.57	1.02	3.06	1.10	0.134
10	People approaching the end of life are offered comprehensive assessments in response to their changing needs and preferences.	R	4.33	0.89	4.16	1.00	0.387
		F	4.02	0.91	3.76	1.08	0.256
		A	3.55	1.07	3.26	1.12	0.514
11	Families and carers of people approaching the end of life are offered comprehensive assessments in response to their changing needs and preferences.	R	4.17	0.94	4.14	0.93	0.824
		F	3.67	1.10	3.56	1.07	0.664
		A	3.36	1.12	3.00	1.25	0.488
12	The assessments made by the interdisciplinary team are continuously monitored.	R	4.29	0.96	4.30	0.97	0.972
		F	3.83	1.13	3.72	1.23	0.763
		A	3.52	1.28	3.28	1.21	0.456
13	People approaching the end of life have their physical needs safely, effectively, and appropriately met at any time of day or night, including access to medicines and equipment.	R	4.38	1.01	4.22	1.17	0.458
		F	3.90	1.09	3.68	1.19	0.363
		A	3.77	1.12	3.42	1.07	0.288
14	People approaching the end of life have their psychological needs safely, effectively, and appropriately met at any time of day or night, including access to medicines and equipment.	R	4.14	1.08	3.94	1.24	0.421
		F	3.48	1.11	3.32	1.27	0.631
		A	3.27	1.15	2.84	1.28	0.246
15	People approaching the end of life are offered social and practical support which is appropriate to their preferences and maximises independence and social participation for as long as possible.	R	4.16	1.04	4.06	1.04	0.561
		F	3.69	1.17	3.52	1.09	0.383
		A	3.52	1.23	3.06	1.11	0.045
16	People approaching the end of life are offered spiritual and religious support appropriate to their needs and preferences.	R	4.19	1.05	4.14	1.01	0.725
		F	3.72	1.29	3.64	1.26	0.659
		A	3.64	1.45	3.42	1.18	0.147
17	Families and carers of people approaching the end of life are offered comprehensive support in response to their changing needs and preferences.	R	4.28	0.93	4.12	0.96	0.313
		F	3.66	0.98	3.66	1.02	0.825
		A	3.59	1.02	3.22	1.09	0.322
18	The team engages with family members in patients’ care.	R	4.41	0.70	4.24	0.96	0.581
		F	4.00	0.82	3.86	1.05	0.656
		A	3.91	0.96	3.52	1.09	0.177
19	People approaching the end of life receive consistent care that is coordinated effectively across all relevant settings and services at any time of day or night.	R	4.40	0.90	4.32	0.89	0.541
		F	3.86	1.05	3.82	1.04	0.808
		A	3.80	1.13	3.30	1.04	0.280
20	People approaching the end of life who experience a crisis at any time of day or night receive prompt, safe, and effective urgent care appropriate to their needs and preferences.	R	4.47	0.90	4.32	0.96	0.345
		F	4.19	1.00	3.86	1.05	0.070
		A	3.98	1.11	3.56	0.99	0.098
21	The team uses clinical care protocols.	R	4.41	0.84	4.38	0.81	0.715
		F	4.05	0.89	4.18	0.90	0.411
		A	3.77	1.01	3.68	1.10	0.855
22	Information about the patient is shared by all professionals involved in the care process.	R	4.50	0.80	4.36	1.06	0.732
		F	4.22	0.86	4.06	1.11	0.643
		A	4.18	0.90	3.78	1.11	0.044
23	There is a procedure and utilisation rules in place for adding information to the clinical record.	R	4.50	0.68	4.44	0.81	0.887
		F	4.26	0.83	4.26	0.75	0.870
		A	4.11	1.02	3.74	0.99	0.362
24	People approaching the end of life receive communication and information in an accessible and sensitive way in response to their needs and preferences.	R	4.38	0.81	4.44	0.88	0.466
		F	4.09	0.92	4.08	1.01	0.883
		A	3.95	1.03	3.54	1.05	0.124
25	Families and carers of people approaching the end of life receive communication and information in an accessible and sensitive way in response to their needs and preferences.	R	4.41	0.75	4.38	0.85	0.992
		F	4.24	0.78	4.12	0.98	0.736
		A	4.18	0.84	3.72	1.07	0.182
26	The professionals on the team safeguard the rights, responsibilities, and safety of the patient.	R	4.50	0.76	4.44	0.79	0.676
		F	4.29	0.82	4.20	0.99	0.817
		A	4.25	0.89	3.88	1.00	0.193
27	The team informs both the patient and his or her legal guardian of the patient’s rights.	R	4.34	0.85	4.22	1.02	0.634
		F	3.93	1.01	3.92	1.18	0.776
		A	3.80	1.15	3.48	1.07	0.217
28	The team has a statement of the rights and guarantees of patients and families available.	R	4.26	0.83	4.16	0.98	0.775
		F	3.88	1.11	3.96	1.03	0.748
		A	3.43	1.23	3.36	1.06	0.821
29	A personalised care plan for people approaching the end of life which is appropriate to their needs and preferences is developed and reviewed.	R	4.33	0.96	4.26	0.88	0.454
		F	3.93	1.02	3.88	1.10	0.895
		A	3.52	1.19	3.26	1.21	0.721
30	The professionals on the team ask the patient and family members how they would like to be informed about the diagnosis/prognosis/treatment progress of the disease and reflect this in the clinical record in a clearly visible place.	R	4.28	0.95	4.20	1.11	0.876
		F	3.88	1.14	3.94	1.20	0.664
		A	3.73	1.26	3.38	1.19	0.100
31	The team enables the patient to be involved in decision-making throughout the course of the disease.	R	4.47	0.92	4.32	1.02	0.272
		F	4.07	1.02	4.02	1.10	0.921
		A	3.91	1.18	3.54	1.22	0.036
32	There is an education programme for patients and families who wish to use it, which facilitates decision-making throughout the course of the disease.	R	3.97	1.15	4.04	1.19	0.633
		F	3.24	1.23	3.46	1.23	0.286
		A	2.41	1.37	2.60	1.34	0.893
33	The team provides information on the benefits and adverse effects of the treatments that may be provided to the patient.	R	4.57	0.75	4.26	1.07	0.107
		F	4.29	0.86	4.10	1.11	0.493
		A	4.07	1.09	3.68	1.10	0.026
34	There is an advance care directive document in place.	R	4.19	1.18	4.14	1.31	0.915
		F	4.05	1.30	3.78	1.47	0.325
		A	2.95	1.55	3.18	1.32	0.894
35	Patient referral criteria are clearly defined.	R	4.33	0.89	4.26	1.03	0.849
		F	3.93	1.11	3.88	1.14	0.831
		A	3.45	1.23	3.40	1.12	0.477
36	People approaching the end of life are identified in a timely manner and receive coordinated care according to a personalised care plan, including prompt access to comprehensive support, equipment, and medication management.	R	4.57	0.73	4.32	1.02	0.247
		F	4.12	0.94	4.02	1.08	0.748
		A	3.95	1.01	3.60	1.12	0.422
37	Protocols and clinical guidelines for providing education and information about the dying phase to the family are available to the team.	R	4.19	1.02	4.20	1.05	0.906
		F	3.72	1.23	3.84	1.11	0.700
		A	3.00	1.35	3.04	1.34	0.410
38	The body of a person who has died is cared for in a culturally sensitive and dignified manner.	R	4.67	0.76	4.52	0.89	0.205
		F	4.62	0.77	4.30	0.97	0.030
		A	4.61	0.84	4.14	1.11	0.066
39	Families and carers of people who have died receive timely verification and certification of the death.	R	4.67	0.60	4.50	0.84	0.379
		F	4.64	0.61	4.50	0.84	0.512
		A	4.59	0.69	4.24	1.08	0.481
40	People closely affected by a death are communicated with in a sensitive way.	R	4.71	0.59	4.44	0.81	0.050
		F	4.52	0.73	4.40	0.83	0.462
		A	4.48	0.88	4.16	0.89	0.068
41	Protocols and clinical guidelines for providing grief care are available to the team.	R	4.24	1.00	4.12	1.02	0.470
		F	3.83	1.13	3.90	1.04	0.837
		A	3.05	1.35	3.02	1.19	0.904
42	Families of the deceased are offered emotional and spiritual support appropriate to their needs and preferences during the grieving process.	R	4.40	0.86	4.22	0.91	0.224
		F	3.93	0.99	3.92	1.03	0.984
		A	3.70	1.17	3.36	1.14	.087

* n = 37 level of attainment; ** n = 27 level of attainment; R: Relevance; F: Feasibility; A: level of attainment. *** Mann–Whitney U-Test.

**Table 3:** Scores given by nursing home (NH) and primary health care (PHC) professionals, according to relevance and feasibility.

			NH *n* = 64		PHC *n* = 44		*p* *	
			M	DS	M	DS		
1	Health and social care workers have the knowledge, skills and attitudes necessary to be competent to provide high-quality care and support for people approaching the end of life and their families and carers.	R	4.34	0.88	4.23	0.91	0.478	
		F	3.97	1.07	3.59	1.04	0.038	NH > PHC
2	Generalist and specialist services providing care for people approaching the end of life and their families and carers have a multidisciplinary workforce sufficient in number and skill mix to provide high-quality care and support.	R	4.23	1.03	4.16	1.10	0.799	
		F	3.50	1.17	3.20	1.13	0.021	NH > PHC
3	The roles and competencies of all professionals on the palliative care team are clearly defined.	R	4.19	0.97	4.32	0.77	0.699	
		F	3.89	1.16	3.68	1.09	0.160	
4	The interdisciplinary team coordinates and works together on case conferences for each of the cases treated by the team.	R	4.16	0.98	4.14	1.03	0.995	
		F	3.77	1.21	3.30	1.23	0.038	
5	The criteria for referral to other professionals in the team/centre are clearly defined: criteria for care by the psychologist, criteria for care by the social worker, criteria for care by the counsellor or spiritual guide.	R	4.23	0.92	4.16	0.94	0.610	
		F	3.91	1.06	3.39	1.19	0.021	NH > PHC
6	The clinical material and medication needed to carry out care work are available to staff.	R	4.22	1.03	4.48	0.85	0.240	
		F	3.67	1.22	4.05	0.86	0.160	
7	The meeting rooms, offices, etc. necessary for carrying out care activities are available to staff.	R	4.52	0.80	4.23	0.89	0.050	
		F	4.41	0.87	4.00	1.06	0.025	NH > PHC
8	People approaching the end of life are identified in a timely manner.	R	4.41	0.81	4.36	0.97	0.907	
		F	4.20	0.89	3.77	1.05	0.030	NH > PHC
9	People approaching the end of life who may benefit from specialist palliative care are offered this care in a timely manner appropriate to their needs and preferences, at any time of day or night.	R	4.30	0.95	4.27	1.00	0.992	
		F	3.77	1.07	3.50	1.02	0.144	
10	People approaching the end of life are offered comprehensive assessments in response to their changing needs and preferences.	R	4.25	0.96	4.25	0.92	0.873	
		F	4.05	0.97	3.68	1.01	0.051	NH > PHC
11	Families and carers of people approaching the end of life are offered comprehensive assessments in response to their changing needs and preferences.	R	4.08	1.01	4.27	0.79	0.459	
		F	3.75	1.05	3.43	1.11	0.135	
12	The assessments made by the interdisciplinary team are continuously monitored.	R	4.38	0.92	4.18	1.02	0.295	
		F	4.00	1.08	3.45	1.23	0.016	NH > PHC
13	People approaching the end of life have their physical needs safely, effectively, and appropriately met at any time of day or night, including access to medicines and equipment.	R	4.25	1.10	4.39	1.06	0.454	
		F	3.86	1.17	3.70	1.09	0.360	
14	People approaching the end of life have their psychological needs safely, effectively and appropriately met at any time of day or night, including access to medicines and equipment.	R	4.06	1.18	4.02	1.13	0.713	
		F	3.56	1.14	3.18	1.23	0.097	
15	People approaching the end of life are offered social and practical support which is appropriate to their preferences and maximises independence and social participation for as long as possible.	R	4.14	1.02	4.07	1.07	0.705	
		F	3.89	1.03	3.20	1.17	0.002	NH > PHC
16	People approaching the end of life are offered spiritual and religious support appropriate to their needs and preferences.	R	4.22	1.00	4.09	1.07	0.543	
		F	4.00	1.11	3.23	1.36	0.003	NH > PHC
17	Families and carers of people approaching the end of life are offered comprehensive support in response to their changing needs and preferences.	R	4.17	1.00	4.25	0.87	0.860	
		F	3.84	0.96	3.39	0.99	0.012	NH > PHC
18	The team engages with family members in patients’ care.	R	4.31	0.81	4.36	0.87	0.576	
		F	4.00	0.94	3.84	0.91	0.339	
19	People approaching the end of life receive consistent care that is coordinated effectively across all relevant settings and services at any time of day or night.	R	4.38	0.90	4.34	0.89	0.730	
		F	4.05	1.00	3.55	1.04	0.010	NH > PHC
20	People approaching the end of life who experience a crisis at any time of day or night receive prompt, safe and effective urgent care appropriate to their needs and preferences.	R	4.39	0.95	4.41	0.90	0.928	
		F	4.08	1.06	3.98	1.00	0.466	
21	The team uses clinical care protocols.	R	4.34	0.91	4.48	0.66	0.769	
		F	4.20	0.95	3.98	0.79	0.089	
22	Information about the patient is shared by all professionals involved in the care process.	R	4.38	0.98	4.52	0.85	0.366	
		F	4.20	1.06	4.07	0.87	0.201	
23	There is a procedure and utilisation rules in place for adding information to the clinical record.	R	4.41	0.79	4.57	0.66	0.347	
		F	4.25	0.82	4.27	0.76	0.978	
24	People approaching the end of life receive communication and information in an accessible and sensitive way in response to their needs and preferences.	R	4.36	0.82	4.48	0.88	0.284	
		F	4.22	0.92	3.89	0.99	0.068	
25	Families and carers of people approaching the end of life receive communication and information in an accessible and sensitive way in response to their needs and preferences.	R	4.41	0.77	4.39	0.84	0.952	
		F	4.30	0.79	4.02	0.98	0.160	
26	The professionals on the team safeguard the rights, responsibilities and safety of the patient.	R	4.47	0.76	4.48	0.79	0.851	
		F	4.36	0.80	4.09	1.01	0.175	
27	The team informs both the patient and his or her legal guardian of the patient’s rights.	R	4.28	0.98	4.30	0.85	0.836	
		F	4.05	1.08	3.75	1.08	0.110	
28	The team has a statement of the rights and guarantees of patients and families available.	R	4.11	0.98	4.36	0.75	0.249	
		F	3.95	1.05	3.86	1.11	0.700	
29	A personalised care plan for people approaching the end of life which is appropriate to their needs and preferences is developed and reviewed.	R	4.25	0.96	4.36	0.87	0.569	
		F	4.02	1.02	3.75	1.10	0.202	
30	The professionals on the team ask the patient and family members how they would like to be informed about the diagnosis/prognosis/treatment progress of the disease and reflect this in the clinical record in a clearly visible place.	R	4.13	1.08	4.41	0.92	0.162	
		F	3.98	1.15	3.80	1.19	0.361	
31	The team enables the patient to be involved in decision-making throughout the course of the disease.	R	4.30	1.03	4.55	0.85	0.178	
		F	4.13	1.06	3.93	1.04	0.176	
32	There is an education programme for patients and families who wish to use it, which facilitates decision-making throughout the course of the disease.	R	3.83	1.23	4.25	1.04	0.067	
		F	3.34	1.25	3.34	1.22	0.990	
33	The team provides information on the benefits and adverse effects of the treatments that may be provided to the patient.	R	4.41	0.94	4.45	0.90	0.881	
		F	4.27	0.98	4.11	0.99	0.298	
34	There is an advance care directive document in place.	R	3.97	1.33	4.45	1.02	0.042	PHC > NH
		F	3.73	1.46	4.20	1.23	0.091	
35	Patient referral criteria are clearly defined.	R	4.22	1.03	4.41	0.82	0.479	
		F	3.97	1.17	3.82	1.04	0.306	
36	People approaching the end of life are identified in a timely manner and receive coordinated care according to a personalised care plan, including prompt access to comprehensive support, equipment, and medication management.	R	4.41	0.92	4.52	0.82	0.514	
		F	4.08	1.06	4.07	0.93	0.717	
37	Protocols and clinical guidelines for providing education and information about the dying phase to the family are available to the team.	R	4.06	1.13	4.39	0.84	0.162	
		F	3.73	1.25	3.84	1.06	0.855	
38	The body of a person who has died is cared for in a culturally sensitive and dignified manner.	R	4.69	0.73	4.48	0.93	0.212	
		F	4.66	0.74	4.20	1.00	0.005	NH > PHC
39	Families and carers of people who have died receive timely verification and certification of the death.	R	4.67	0.62	4.48	0.85	0.259	
		F	4.67	0.62	4.43	0.85	0.090	
40	People closely affected by a death are communicated with in a sensitive way.	R	4.64	0.65	4.50	0.79	0.315	
		F	4.61	0.68	4.25	0.87	0.014	NH > PHC
41	Protocols and clinical guidelines for providing grief care are available to the team.	R	4.17	1.00	4.20	1.02	0.791	
		F	4.00	0.98	3.66	1.20	0.164	
42	Families of the deceased are offered emotional and spiritual support appropriate to their needs and preferences during the grieving process.	R	4.36	0.76	4.25	1.04	0.934	
		F	4.17	0.85	3.57	1.11	0.004	NH > PHC

R: Relevance; F: Feasibility. * Mann–Whitney U-Test.

## Data Availability

The data presented in this study are available on reasonable request from the corresponding author. The data are not publicly available due to privacy restrictions.
